# Impact of disabled circadian clock on yellow fever mosquito *Aedes aegypti* fitness and behaviors

**DOI:** 10.1038/s41598-022-10825-5

**Published:** 2022-04-27

**Authors:** Vinaya Shetty, Jacob I. Meyers, Ying Zhang, Christine Merlin, Michel A. Slotman

**Affiliations:** 1 Department of Entomology, Texas A&M University, College Station, TX 77843 USA; 2 Department of Biology, Texas A&M University, College Station, TX 77843 USA

**Keywords:** Genetics, Molecular biology, Zoology, Behavioural ecology

## Abstract

Like other insects, *Aedes aegypti* displays strong daily patterns in host seeking and mating. Much of these behaviors are believed to be under the control of a circadian clock, an endogenous timekeeping mechanism relying on transcriptional/translational negative feedback loops that drive rhythmic physiology and behavior. To examine the connection between the circadian clock and various *Ae. aegypti* behaviors, we knocked out the core clock gene *cycle* using CRISPR/Cas9*.* We found that the rhythmic pattern and intensity of mRNA expression of seven circadian genes, including *AeCyc*^*−/−*^, were altered across the day/night cycle as well as in constant darkness conditions. We further show that the mutant CYC protein is incapable of forming a dimer with CLK to stimulate *per* expression and that the endogenous clock is disabled in *AeCyc*^*−/−*^ mosquitoes. *AeCyc*^*−/−*^ do not display the bimodal locomotor activity pattern of wild type, have a significantly reduced response to host odor, reduced egg hatching rates, delayed embryonic development and reduced adult survival and mating success. Surprisingly however, the propensity to blood feed in *AeCyc*^*−/−*^ females is significantly higher than in wildtype females. Together with other recent work on the circadian clock control of key aspects of mosquito biology, our data on how *cycle* KO affects mosquito behavior and fitness provides a basis for further work into the pathways that connect the mosquito endogenous clock to its vector competence.

## Introduction

Eukaryotic organisms have endogenous 24-h internal circadian clocks that assist them in optimizing their physiology and behavior to daily fluctuations in light, temperature, and resource availability^1^. This has allowed many organisms to adapt to a temporal niche, displaying certain behaviors only during specific times of the light–dark (LD) cycle. This is true for mosquito disease vectors, in which circadian rhythms in behavior and gene expression is well-documented^2–8^. *Aedes aegypti*, the primary vector of numerous emerging vector-borne diseases including Yellow Fever, Dengue, Chikungunya, and more recently Zika virus^9^ displays a small activity peak at the start of the light phase but is primarily active during the late afternoon hours^10^. The diurnal activity patterns of *Aedes aegypti* are found in flight activity, oviposition, host-seeking, and human landing/biting^11^. The endogenous circadian clock of mosquitoes regulates locomotor activity and blood feeding behavior^11–13^, and is expected to regulate the timing of other behaviors such as host-seeking, mating and oviposition, although this has not been specifically tested^14^.

The mosquito circadian clock, which is entrained by the light:dark cycle, relies on two interlocked transcriptional/translational negative feedback loops that cycle every 24 h^14^. The core circadian genes at the center of this feedback loop are *cycle*, *clock*, *period*, *timeless*, *pdp1*, and *cryptochrome-2*^5,6,15,16^. In some insects, e.g. the monarch butterfly, *cycle* is now referred to as *bmal1*^17^. This is because in these species, as in mosquitoes, *cycle* retains a C-terminal transactivation domain that is present in the mammalian orthologue *bmal1* but is missing in the Drosophila orthologue *cycle*. Here, we refer to *Aedes aegypti cycle (AeCyc)* for consistency with the current genome annotation^18^. In addition to sustaining 24-h rhythms, the circadian clock also regulates the rhythmic expression of a broad range of genes that drive circadian behaviors^19–22^. In *Anopheles gambiae* it was demonstrated that circadian dependent modulation of olfactory responses significantly influences the distinct behavioral responses in mosquitoes^23^.

However, much less is known about how molecular clock disruption affects critical sensory and motor systems in mosquito disease vectors^14^. RNAi-mediated knockdown of *timeless* in *Ae. aegypti* caused a decrease in locomotor activity and an increase in the free-running period^13^, but no other behavioral assays were performed in this study. In *Anopheles gambiae,* knocking down *timeless* or *cryptochrome 1* using RNAi increases blood feeding propensity^12^. Furthermore, the expression of these genes was affected by short light pulses^12^. In *Ae. aegypti* blood feeding reduces expression of the four critical clock genes *clock, cycle, timeless*, and *period*^13^. Female mosquitoes stop responding to host cues after blood-feeding, and it has been hypothesized that suppression of endogenous clock genes is the mechanism through which this is achieved^24^. These studies demonstrate a connection between circadian clock genes and blood feeding behavior. Whether this is the case for other mosquito behaviors has not been established.

Given the pervasive regulation of Drosophila behaviors by the circadian clock and the conserved nature of the circadian clock between flies and mosquitoes^14^, a functional circadian clock is likely critical to the regulation of many mosquito behaviors as well. Studying genes controlling host-seeking and other behaviors is important, as they may be potential targets for future vector control^25^. To explore the connection between the endogenous clock and various mosquito behaviors, we used CRISPR/Cas9-mediated gene knockout of *AeCycle* to disable the endogenous circadian clock in *Aedes aegypti.* Disruption of the clock abolishes the characteristic circadian locomotor activity patterns and significantly reduces *Ae. aegypti’s* response to human host odor, mating success, but not blood feeding. Furthermore, it also delays larval development and reduces adult life span. These data show the pivotal role of the endogenous circadian clock in the biology of *Ae. aegypti* and opens the door to future work fully characterizing the function and role of the circadian clock in this important human disease vector.

## Results

### Generation of a ***AeCyc***^***−/−***^ strain

**Figure 1:**
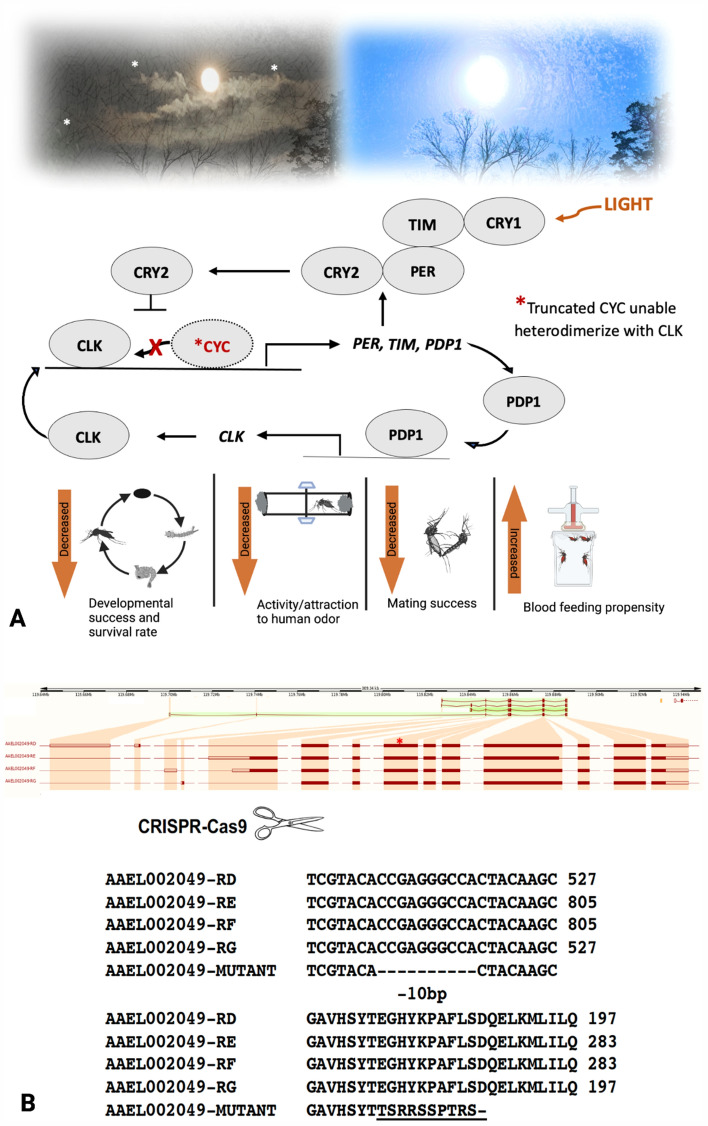
(**A**) Schematic illustration of the molecular mechanism of circadian rhythms at day/night cycle. AeCYC knockout is unable to bind with AeCLK and initiate the transcription of downstream genes in the pathway. *AeCyc*^*−/−*^ impacts on its developmental stages and other behaviors. (**B**) CRISPR/Cas9-mediated deletion of 10 bp from the exon 5 of *cycle* gene in *Ae. aegypti*. AAEL002049-RD, RE, RF and RG are four alternative splice variants, and deletion of 10 bp from the exon 5 causing a frame shift, resulted in a premature stop codon in all the four gene splice variants. *Indicates the location of deletion in exon 5 (AAEL002049-RD).

CRISPR/Cas9-mediated targeted mutagenesis was used to generate a mutant bearing a 10 bp deletion in exon 5 of *AeCyc* (Fig. 1A,B). This deletion, in addition to causing a frame shift, resulted in a premature stop codon 30 bp upstream from the deletion. Any resulting protein would therefore include only the first 177 out of 767 CYCLE (CYC) amino acids, followed by 11 additional amino acids. Importantly, the truncated protein would contain the two helices necessary for DNA binding domain, but neither the transactivation domain, nor the two PAS domains that are thought to be responsible for dimerization with CLOCK. The F_1_ mutant of this strain was backcrossed to wildtype *Ae aegypti* for four generations before a homozygous *AeCyc*^*−/−*^ strain was created.

### Endogenous clock gene expression

**Figure 2:**
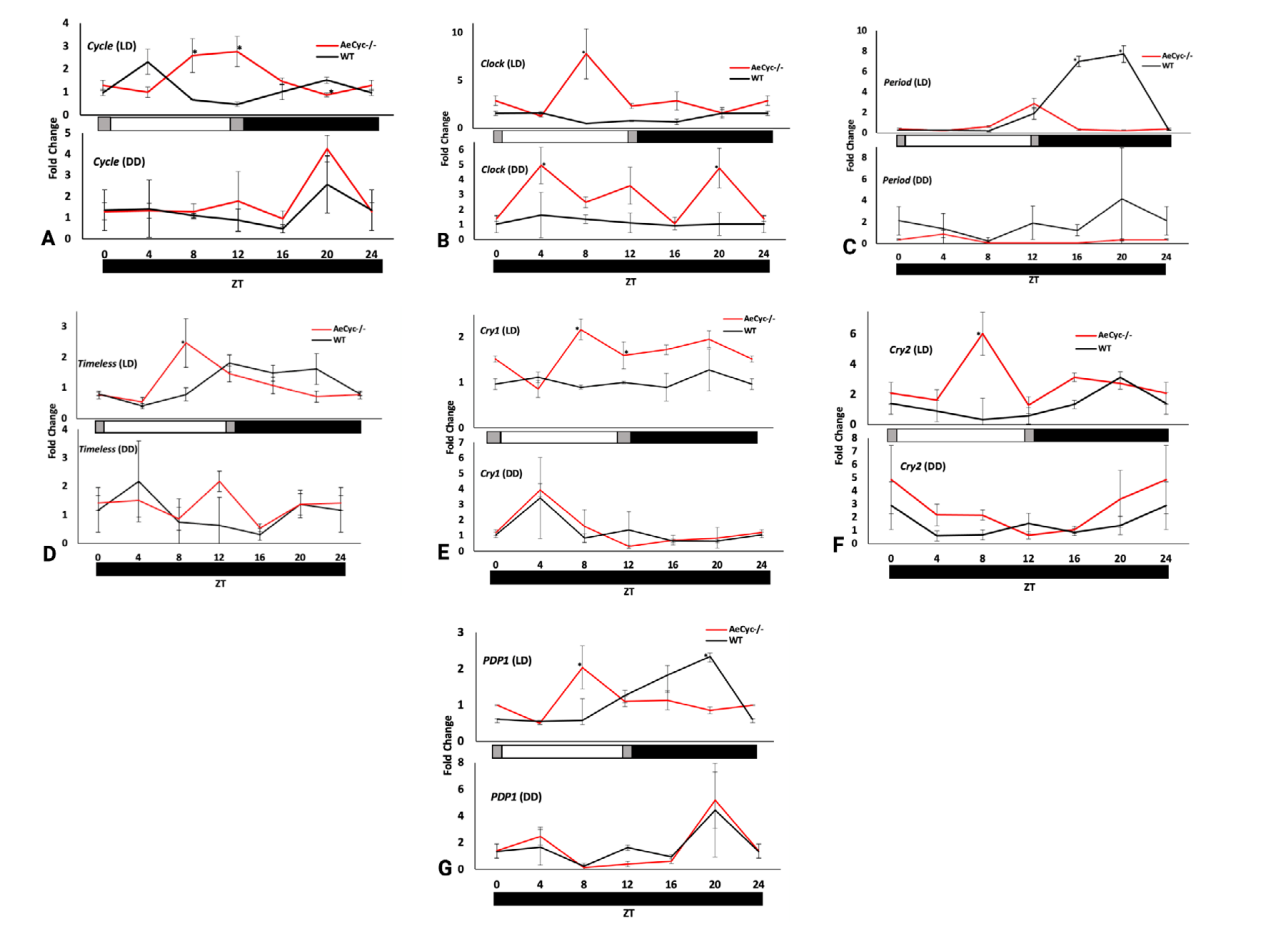
Gene expressions of (**A**) *cycle*, (**B**) *clock*, (**C**) *per*, (**D**) *tim*, (**E**) *cry1*, (**F**) *cry2*, (**G**) *pdp1* in *AeCyc*^*−/−*^ and wildtype mosquitoes at different time points across LD and DD cycles. Relative mRNA expression measured by quantitative real-time PCR. Each timepoint represents the average of three biological replicates (n = 3). Black bars = light off, white bars = bright light, dark gray bars = dim light. ‘*’ indicates statistically significant differences (p ≤ 0.05) in peaks between *AeCyc*^*−/−*^ and wildtype at each time points.

Next, we examined the impact of *Cyc* KO on the relative expression and rhythmic appearance of seven essential circadian clock genes: *AeCyc*, *AeClk*, *AePer*, *AeTim*, *AeCry1*, *AeCry2* and *AePdp1* at 4 h intervals in light:dark conditions (ZT0 to ZT24). The mRNA expression study confirmed that the cyclical expression pattern of six clock genes is altered in *AeCyc*^*−/−*^, with the timing of peak expression shifted to different time points (Fig. 2A–G). Somewhat surprisingly, *AeCyc*^*−/−*^ mRNA was detected, and expression of this mutant gene did exhibit a cyclical expression pattern under LD conditions, with similar amplitude as in the wildtype strain but in antiphase. Interestingly, the cyclical expression pattern was similar between wildtype and *AeCyc*^*−/−*^ under DD conditions (Fig. 2A).

The other endogenous clock genes, with the exception of *AeCry1*, also showed a shift in the timing of peak expression under LD conditions (Fig. 2A–G). For *AeCry1* the pattern was not very clear. Furthermore, while the expression of *AeClk* was enhanced in *AeCyc*^*−/−*^ under both LD and DD conditions, expression of *AePer* was much reduced in *AeCyc*^*−/−*^*.* In addition, *AeCry1* and *AePdp1* showed a marked peak in expression in both strains under DD conditions at ZT4 and ZT20, respectively. Overall, the changed cyclical expression pattern of these genes points towards a non-functional or disabled endogenous clock in *AeCyc*^*−/−*^.

### Transcription assay

**Figure 3:**
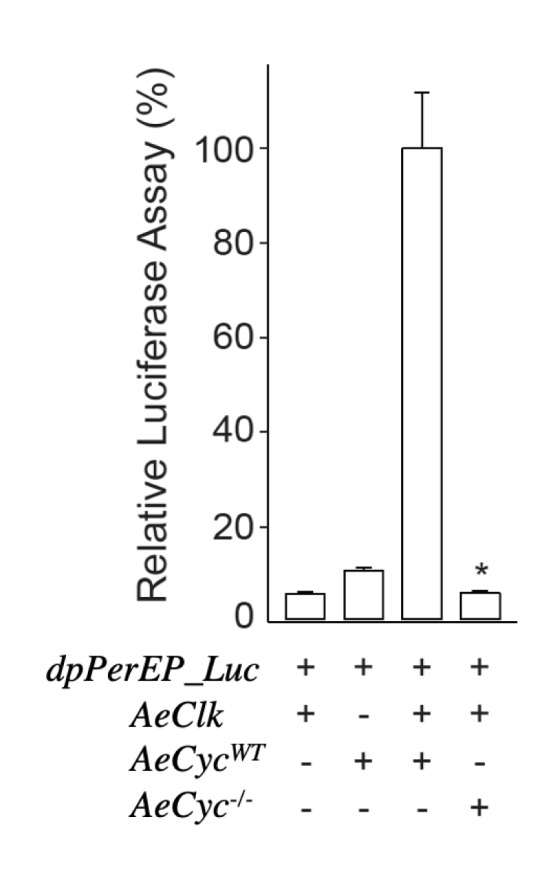
The mutant *AeCyc* lacking the PAS and transactivation domains is impaired in transcriptional activation in S2 cells. The monarch *per* E box luciferase reporter (dpPerEp_Luc; 10 ng) was used in presence (+) or absence (–) of wildtype *AeClk*, *AeCyc*^*WT*^, *AeCyc*^*−/−*^ expression plasmids (5 ng each). Firefly luciferase activity was computed relative to renilla luciferase activity. Each value is mean ± SEM of three replicates. One-way ANOVA, AeCLK:AeCYC^*−/−*^ vs. AeCLK:AeCYC^WT^, *p ≤ 0.002.

Because mutant *AeCyc*^*−/−*^ mRNA is expressed in *AeCyc*^*−/−*^ mosquitoes, we verified that AeCLK:AeCYC^*−/−*^ did not activate transcription in *Drosophila* schneider 2 (S2) cells co-expressed with monarch butterfly per E-boxes driving luciferase as a reporter, while AeCLK:AeCYC^WT^ does activate transcription (one-way ANOVA, *p* < 0.002) (Fig. 3). This is as expected if the truncated CYC protein was lacking the PAS domains necessary for dimerization with CLOCK. The control experiment also showed that AeCLK does not bind to endogenous *Drosophila melanogaster* DmCYC to activate luciferase transcription. These data indicate that *AeCyc*^−/−^ is non-functional, which should inactivate the endogenous circadian clock.

### Circadian locomotor activity

**Figure 4:**
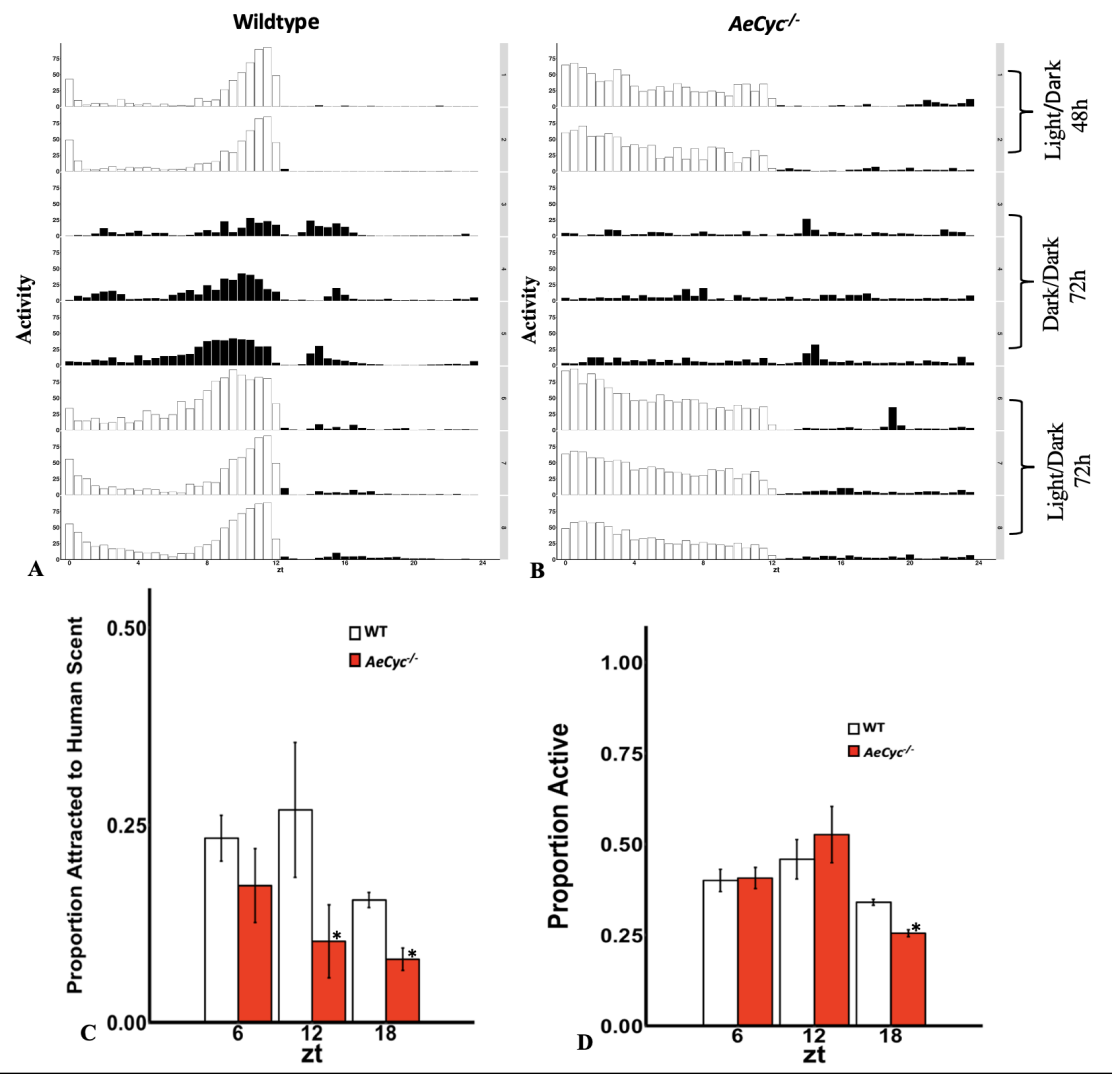
(**A**, **B**) Circadian activity patterns in wildtype and *AeCyc*^*−/−*^ mutants in both both LD and DD conditions. (**C**) Attraction of wildtype and *AeCyc*^*−/−*^ females to human odor in olfactometer. Proportion attracted = the proportion of mosquitoes in the arm of the olfactometer that contained the sock. (**D**) Comparing activation of wildtype and *AeCyc*^*−/−*^ mutants in olfactometer. “activation” = the proportion of females leaving the release cage of olfactometer. *Statistically significant p ≤ 0.05.

To further confirm disruption of the circadian clock in *AeCyc*^*−/−*^, we compared circadian flight activity patterns between wildtype and *AeCyc*^−/−^ in a locomotor activity monitor. The movement of each mosquito was detected by the interruption of an infrared sensor on the monitor. Wildtype mosquitoes show the expected circadian activity pattern, with a small peak of activity at the start of the light phase (ZT0), and a pronounced activity peak towards the end of the light phase (ZT8-ZT12, Fig. 4A). During the dark phase (ZT12-ZT24) little activity was observed. During 72 h under DD conditions, the main activity peak between ZT8 and ZT12 was maintained, with an expected shift towards an earlier pattern of LD conditions (Fig. 4A). In contrast, *AeCyc*^*−/−*^ mosquitoes show constant activity throughout the light phase, with an overall significantly higher activity (74.5 beam breaks/hour on day 1 + 2) during this period compared to wildtype (41.6 beam breaks/hour on day 1 + 2, p < 0.001, Fig. 4B). Similarly to wildtype, *AeCyc*^*−/−*^ mosquitoes become largely inactive during the dark phase (5.4 beam breaks/hour, p = 0.731).

Importantly, in contrast to wildtype, *AeCyc*^*−/−*^ do not maintain a circadian locomotor activity pattern under DD conditions, showing low overall activity levels (10.8 beam breaks/hour) between ZT0 and ZT12 without any defined peak. Specifically, the level of activity between ZT0 to ZT12 is similar to that between ZT12 to ZT24 (10.6 beam breaks/hour). Furthermore, the level of activity displayed by *AeCyc*^*−/−*^ between ZT0 and ZT12 under DD conditions (10.8 beam breaks/hour) is significantly less than that of wildtype mosquitoes (28.4 beam breaks/hour, p < 0.0001). After restoring LD conditions following 72 h of DD conditions, both wildtype and *AeCyc*^*−/−*^ revert back to their respective LD activity patterns.

### Life history parameters

The hatching rate for *AeCyc*^*−/−*^ was significantly lower than for wildtype *Ae. aegypti,* (0.55 ± 0.18 vs. 0.90 ± 0.12, p = 0.0038, Table 1). In addition, the average egg development time was significantly longer in *AeCyc*^*−/−*^ vs. wildtype eggs (7.12 ± 4.51 vs. 2.03 ± 0.04, t = − 2.7663, df = 5.0006, p = 0.039). Furthermore, fewer hatched larvae survived to the pupal stage in *AeCyc*^*−/−*^ vs. WT (0.93 ± 0.06 vs. 0.99 ± 0.01, p = 0.038), although this difference was small compared to the hatching rates. In contrast to egg development, we found no significant difference between the larval development times between *AeCyc*^*−/−*^ and wildtype (5.27 ± 0.13 vs. 5.23 ± 0.28, t = − 0.30653, df = 7.217, p = 0.767).

**Table 1:** Comparison of life history traits between *AeCyc*^*−/−*^ and wildtype *Ae. aegypti* mosquitoes.

	Genotype	Sex	Success rate (mean ± SD)	Average day of success (mean ± SD)	Time to 50% success (LT_50_, 95% CI)
Egg to larva	Wildtype		0.90 ± 0.12	2.03 ± 0.04	1.58 [1.21, 1.97]
	*AeCyc*^*−/−*^		0.55 ± 0.18*	7.12 ± 4.51*	3.72 [1.79, 5.23]
Larva to pupa	Wildtype		0.99 ± 0.01	5.23 ± 0.28	4.73 [4.65, 4.81]
	*AeCyc*^*−/−*^		0.93 ± 0.06*	5.27 ± 0.13	4.79 [4.71, 4.87]
Pupa to adult	Wildtype	Male	1.00	1.36 ± 0.09	0.95 [0.94, 0.97]
	*AeCyc*^*−/−*^		0.99 ± 0.01	1.50 ± 0.04	1.20 [1.08, 1.32]
	Wildtype	Female	0.99 ± 0.01	1.66 ± 0.08	0.94 [0.92, 0.96]
	*AeCyc*^*−/−*^		0.98 ± 0.01	1.68 ± 0.15	1.18 [1.07, 1.29]

*Statistically significant p ≤ 0.05.

**Figure 5:**
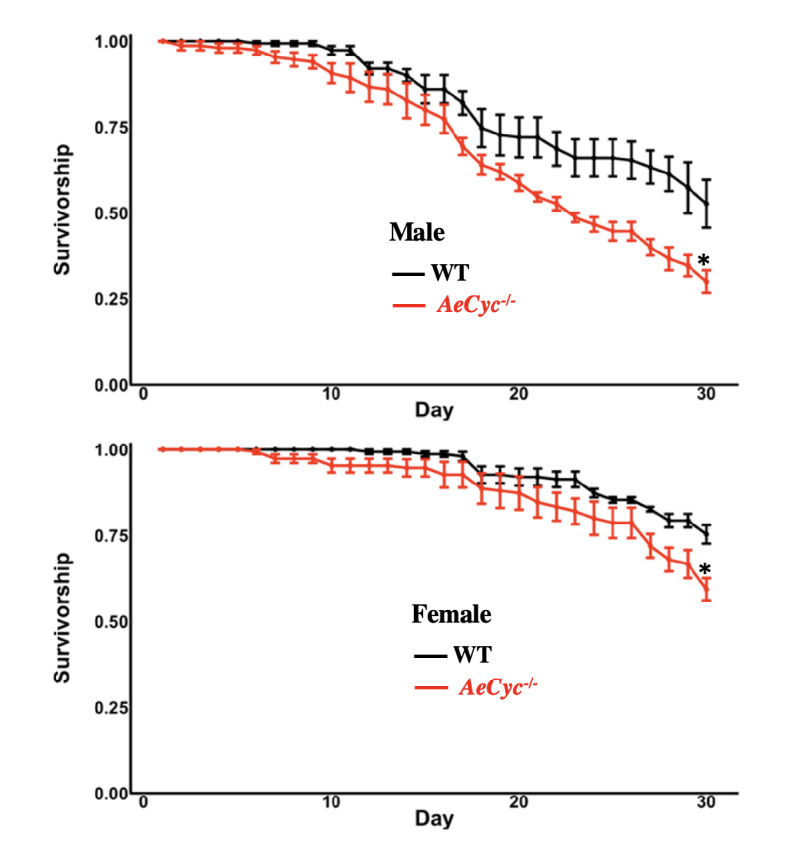
Survivorship of *AeCyc*^*−/−*^ and wildtype *Ae. aegypti* males and females. *Statistically significant p ≤ 0.05.

The adult emergence rate did not differ significantly different between *AeCyc*^*−/−*^ and wildtype *Ae. aegypti* (t = 0.8584, df = 10.008, p = 0.4108), but the survivorship of male and female *AeCyc*^*−/−*^ adults was significantly lower than that of wildtype over a 30-day period (Fig. 5). Male *AeCyc*^*−/−*^ were nearly twice as likely to die during this period than wildtype males (Hazard Ratio: 1.95 [1.28, 2.95], p < 0.001). Female *AeCyc*^*−/−*^ had a significant similar reduction in survivorship (Hazard Ratio: 1.821 [1.346, 2.465], p = 0.00171).

### Mating success

**Figure 6:**
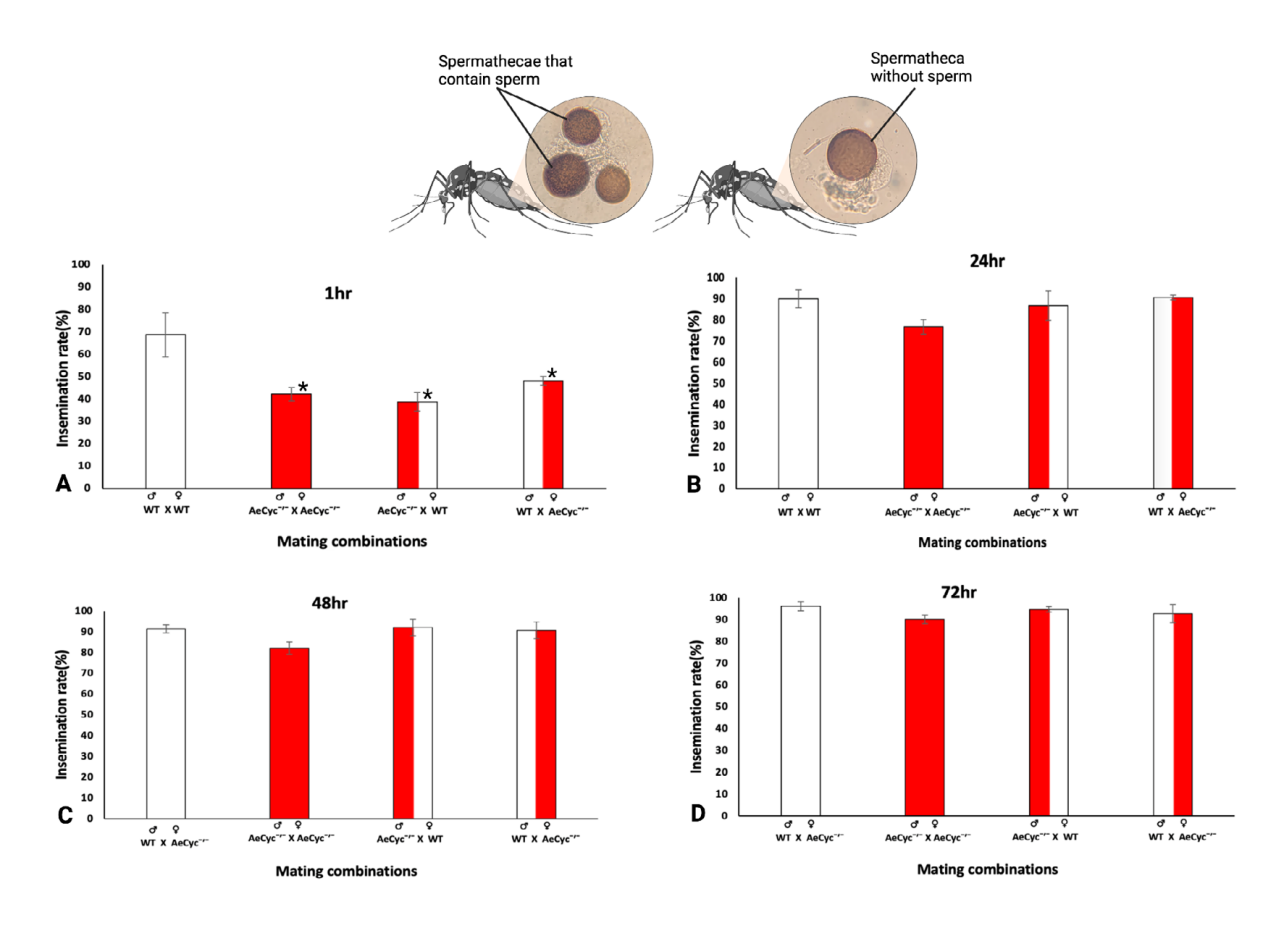
Insemination status of *AeCyc*^*−/−*^ (♂ &♀) and wildtype (♂ &♀) (Nonreciprocal) crossing, and reciprocal crossing groups *AeCyc*^*-/*-^♂ and WT♀; *AeCyc*^*−/−*^♀ and WT ♂ mosquitoes after 1 h (**A**), 24 h (**B**), 48 h (**C**) and 72 h (**D**) of exposure to males for mating (copulation duration). Each value is mean ± SD of three replicates. *Statistically significant p ≤ 0.05.

The mating success of *AeCyc*^*−/−*^ and wildtype *Ae. aegypti* was assessed by determining insemination rates of five-day old virgin mosquitoes. Mating success was significantly lower in *AeCyc*^*−/−*^ mosquitoes than in wildtype after 1 h of female-to-male exposure (42.0% vs 68.7%, p < 0.005, Fig. 6A). The insemination rate was not significant lower in *AeCyc*^*−/−*^ females in the 24 h, 48 h and 72 h exposure experiments (Fig. 6B–D). Interestingly, the insemination rate following 1 h of male exposure was significantly lower in both reciprocal crosses than in wildtype crosses (48.0% vs 68.7%, p = 0.003 for ♀_*cyc*_^*−/−*^/♂_wt_; and 38.7% vs 68.7%, p = 0.002 for ♀_wt_ /♂_*cyc*_^*−/−*^). No significant differences were observed after 24 h, 48 h or 72 h exposure. We also did not observe any differences between the two reciprocal crosses. Furthermore, insemination rates increased significantly in both *AeCyc*^*−/−*^ and wildtype matings as time of exposure to males increased from 24 to 72 h (Huynh–Feldt: df = 2, F = 19.43, p = 0.001). Finally, although we did not quantify this measure, we note that *AeCyc*^*−/−*^ females appeared to store less sperm, most of the sperm was only in one or two of the three spermathecal capsules.

### Attraction to human odor

Next, we compared the attraction of *AeCyc*^*−/−*^ and wildtype females to human odor (white worn socks) in a dual choice olfactometer assay at three different time points, ZT6, ZT12 and ZT18. ZT6 is in the middle of the light phase when wildtype females show low activity levels, ZT12 is just prior to the transition to the dark phase, when wildtype females are highly active, and ZT18 is in the middle of the dark phase. The sock was placed in one of the two collecting chambers connected to the odor ports in olfactometer, and the other collecting chamber was left empty.

Both *AeCyc*^*−/−*^ and wildtype females show some response to host odor at each of the three time points (Fig. 4C). Interestingly, *AeCyc*^*−/−*^ females are significantly less attracted to human odor than wildtype females at ZT12 and ZT18 (p = 0.001 and p = 0.022, respectively, Fig. 4C). These p-values remain significant following Holms-Bonferroni correction. *AeCyc*^*−/−*^ females’ odor response was also lower at ZT6, but this difference was not significant. Furthermore, wildtype females are significantly more attracted to human odor at ZT12 than at ZT18 (p = 0.013). *AeCyc*^*−/−*^ females appear to respond more to human odor at ZT6 than at ZT12 and ZT18 but the difference was not significant following Holms-Bonferroni correction.

### Activation in olfactometer

To examine if differences in response to human odor can be explained by different activity levels of *AeCyc*^*−/−*^ and wildtype females, we also recorded the proportion of females leaving the release cage of olfactometer (= “activation”). During ZT6 and ZT12 the activation rate of *AeCyc*^*−/−*^ and wildtype females was similar, with slightly higher activation in *AeCyc*^*−/−*^ females (p > 0.005, Fig. 4D). Therefore, the lower odor response of *AeCyc*^*−/−*^ females at ZT6 and ZT12 is not due to a lower activation rate. At ZT18, the activation rate was slightly smaller in *AeCyc*^*−/−*^ females (Fig. 4D, p = 0.049), a difference that was not significant after Holms-Bonferroni correction. Not surprisingly, activity of both wildtype and *AeCyc*^*−/−*^ females was significantly higher during the light phase (ZT6 and ZT12) compared to the dark phase (ZT18) (p < 0.005).

### Blood feeding activity

**Figure 7:**
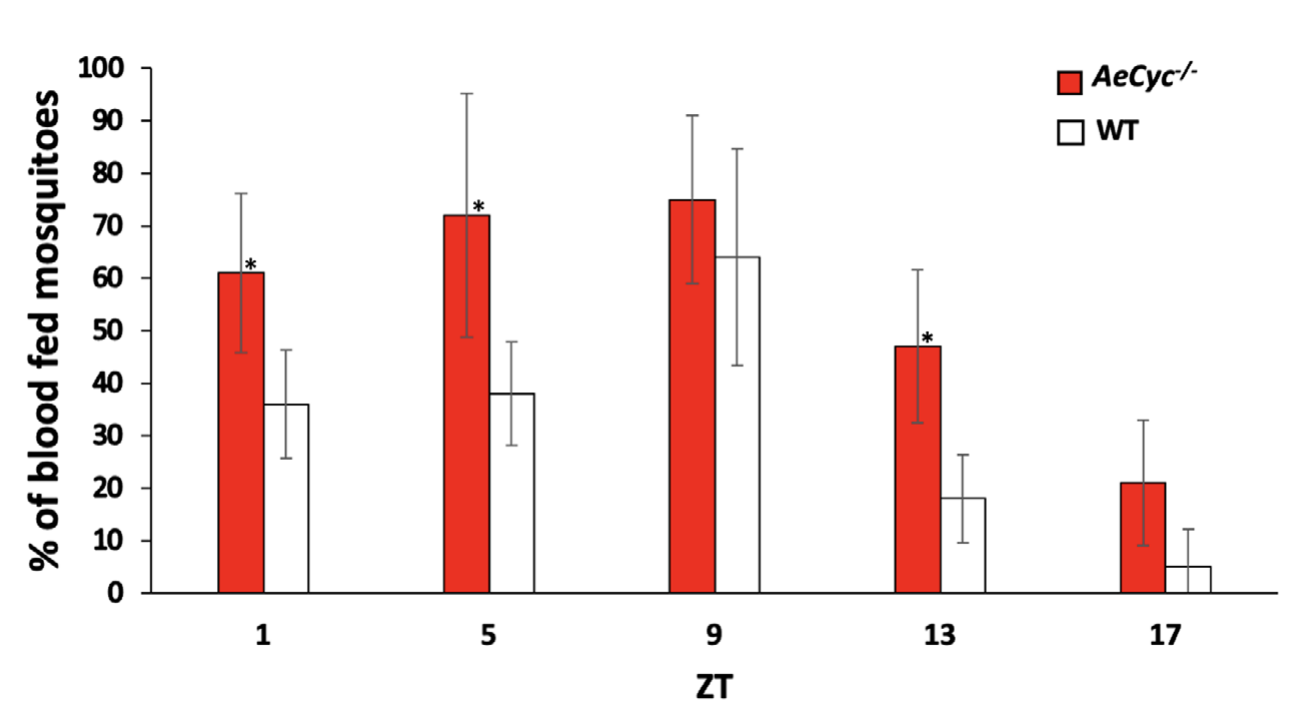
*AeCyc*^*−/−*^ and wildtype *Ae. aegypti* blood-feeding behavior. Mosquitoes were allowed to feed for 20 min and a total percentage of blood fed mosquitoes (includes partially and fully fed) in each time point was scored and plotted. Error bars indicate the standard error. *Statistically significant p ≤ 0.05.

To study the impact of *cycle* KO on blood feeding*,* we performed a time course analysis of blood feeding propensity during the LD cycle. The blood feeding propensity of *AeCyc*^*−/−*^ females peaked during daytime compared to night and somewhat surprisingly, was significantly (p > 0.005) higher than that of wildtype females at ZT1, ZT5, ZT13 and ZT17 (Fig. 7, Table S3). At ZT9 however, when both have the highest feeding propensity, the difference was not significant (p = 0.10), although the trend was the same (*AeCyc*^*−/−*^: 75% vs. WT: 64%).

## Discussion

Knocking out the *cycle* gene in *Aedes aegypti* disables its endogenous circadian clock, resulting in locomotor activity that is stimulated solely by the presence of light but that does not show the typical morning and evening peaks displayed by wildtypes. When light is present *AeCyc*^*−/−*^ are indeed active throughout the light phase but revert to inactivity in darkness. Somewhat surprisingly, *AeCyc*^*−/−*^ mRNA was detected in *AeCyc*^*−/−*^ heads and did show a cyclical expression pattern. *AeCyc*^*−/−*^ contains a premature stop codon and often such transcripts are removed through the nonsense mediated decay pathway (NMD), a process by which aberrant mRNAs that contain a premature stop codon are degraded^26^. However, we demonstrated that if any AeCYC^−/−^ were produced, it is incapable of forming a functional heterodimer with CLK. In *Drosophila melanogaster*, the CYC/CLK heterodimer is an essential component of the core circadian feedback loop that activates the transcription of both *per* and *tim*^27^*.* Consistent with a recent finding that PER protein levels are much reduced in circadian neuronal circuits of *Ae. aegypti* whose circadian clock is disrupted by exposure to constant light conditions^28^, we did find a much reduced expression of *per* under both LD and DD conditions in heads of *AeCyc*^*−/−*^.

However, the expression level of *tim* was similar between *AeCyc*^*−/−*^and wildtype but showed a different cyclical pattern. Furthermore, a study in Drosophila indicated that the low PER and TIM levels may be due to a lack of CYC:CLK heterodimer transcriptional regulators in the homozygous *Cycle* KO mutant strain^29^.

The cyclical expression of other essential endogenous clock genes was disrupted in *AeCyc*^*−/−*^ females as well. *Clk* expression was higher in *AeCyc*^*−/−*^ compared to wildtype under both LD and DD conditions. In contrast, the expression of *Cry1,* which is sensitive to light and inhibits the formation of PER/TIM heterodimers^30^, was similar between *AeCyc*^*−/−*^ and wildtype under LD and DD conditions. A considerable portion of the transcriptome shows circadian expression patterns^6,8^. Although the direct link between the expression of these and the essential clock genes is not clear, it is expected that disrupting the cyclical expression of endogenous clock genes would impact the expression of a variety of clock-controlled genes.

Studies in insects including Drosophila reported that circadian clock regulates the duration of preadult development^31^. Several earlier studies have shown that pupation is gated in mosquitoes such as *Anopheles gambiae*^32^ and *Aedes taeniorhynchus*^33^, and is regulated by the duration of the photoperiod via the secretion of the prothoracicotropic hormone that stimulates ecdysone secretion^34^. Also, study showed that importance of the light cycle and the *period* gene in developmental time memory specifically pupation and adult emergence in fruit flies *D. melanogaster* is under circadian clock control^35,36^. The environmental cycle and *period* allele both determines the time taken for each pre-adult developmental stages such as time taken for pupation^37^. The significantly longer hatching time and lower hatching rates of *AeCyc*^*−/−*^ indicates that this gene is involved in the proper timing and success of embryogenesis. The lower hatching rate is unlikely to be explained by the slightly lower insemination rate of *AeCyc*^*−/−*^*.* In addition, the pupation rate, and adult emergence is significantly reduced, although much less drastically than the hatching rate. The disruption of the circadian clock also impacts basic fitness parameters in Drosophila. Mutants lacking a functional clock have reduced egg production, as well as a reduced hatching rate^37^. Also, in immature stages of Drosophila (egg, larvae, pupae), null mutations in *period* increase development time under normal light/dark conditions^38^. Together with previous findings in Drosophila, where *period* mutants under prolonged or shortened circadian cycles have different development times from eggs to adults^39^. We also found that adult survival across a 30-day period (days 5 through 35) was reduced significantly, indicating that other developmental periods are also affected by the absence of a functional *AeCyc* as well, although to a lesser degree. Furthermore, *cycle* KO mutants in *Drosophila* also show a decrease in survivorship. Surprisingly however, this decreased fitness was observed only in males and the sex-specific mechanisms driving this phenotypic difference is not understood^40^. This is in contrast to our observation that both males and female *AeCyc*^*−/−*^ have reduced survivorship. A potential explanation could be the difference in chromosomal sex determination between Drosophila and mosquitoes that Drosophila have heterogametic sex chromosomes, whereas *Ae. aegypti* do not.

Importantly, similar *cycle* or *clock* knockouts in *D. melanogaster* resulting in arrhythmic, infrequent locomotor activity^41–43^, *AeCyc*^*−/−*^ also display several behavioral deficiencies compared to wildtype mosquitoes. *AeCyc*^*−/−*^ are active throughout the photophase, without showing the bimodal activity pattern typical of wildtype, and become inactive under dark conditions, even when these are imposed for an extended period. This further demonstrates that *AeCyc*^*−/−*^ no longer have a functioning endogenous circadian clock. While *AeCyc*^*−/−*^ show activity throughout the light phase, their activity level is highest at the start and declines slowly until this phase ends. Possibly this decline is related to diminishing energy reserves across this period of high activity. This activity pattern indicates that light provides a strong stimulus and that the mosquito’s response to this stimulus is strongly modulated by the clock*.* This is consistent with previous work which showed that *Ae. taeniorhynchus* exposed to constant LL conditions, and whose clock therefore has been disrupted, display irregular excessive outburst of activity^44^. Previous work also showed that the temporary RNAi-mediated knockdown of *timeless* in *Ae. aegypti*, one of the central clock genes, caused a temporary decrease in regular diurnal peak activity patterns^13^. Although this RNAi approach had only a transient effect, results appear to be consistent with our observation.

An additional important behavioral deficiency of *AeCyc*^−/−^ females is the 65% reduction in their response to human odor during peak activity hours, despite the fact that both were activated to the same degree in the olfactometer. Olfaction is essential for many behaviors in mosquitoes and other insects (e.g., blood and sugar feeding, mating, and oviposition) and is itself under circadian control in insects^42,45^. In *Ae. aegypti*, antennal sensitivity to some host odors is highest towards the end of the light phase, although for others sensitivity peaks during midday^46^. In *An. gambiae,* odorant binding proteins are thought to be involved in modulating temporal changes in odorant sensitivity, enabling the olfactory system to dictate the circadian niche^7^.

Odor responses in *Drosophila* antennae are controlled autonomously by circadian clocks present within olfactory sensory neurons (OSNs)^42,47^. GPRK2 has been identified as a key regulator of olfactory receptor (OR) function and olfactory responses, and GPRK2 levels are under the control of circadian molecular pacemakers located within the OSNs^44,45^. Several olfaction genes in *Aedes* and *Anopheles* show circadian expression patterns, indicating that they may be under control of the endogenous circadian clock^7,8,46^. Also, a study in *An. gambiae* revealed that circadian dependent expression of antennal proteins involved in modulating of temporal changes in odorant sensitivity^23^. Thus, disrupting the circadian clock should alter the expression of olfactory genes, resulting in impaired odor response. Olfactory responses are likely modulated both by peripheral and centralized processes^46^, and whether the reduced odor response observed here is due to a reduced antennal response or due to higher order processes in the cerebral ganglion remains to be determined. Future studies using RNAseq and electroantennogram analyses will be necessary to answer this question.

In addition to having a reduced response to host odor and impaired locomotor activity, *AeCyc*^*−/−*^ also show a lower mating efficiency when given a short mating opportunity time (i.e. within an hour), which likely reflects their mating opportunity in the wild. Under these conditions, the insemination rate of females in crosses involving both sexes of *AeCyc*^*−/−*^ was reduced between 30.1 and 43.7%. These one-hour mating experiments were performed during the last part of the light phase, when wildtype show peak activity levels but *AeCyc*^*−/−*^ mutants showed lower activity. It is thus possible that this may explain some of the observed differences.

Interestingly, *AeCyc*^*−/−*^ females and males contributed equally to the reduction in the insemination rate, but their effects were not additive. That is, crosses in which only one of the sexes were *AeCyc*^*−/−*^ had a similar insemination rate as crosses in which both males and females belonged to *AeCyc*^−/−^. In addition, we noticed that *AeCyc*^*−/−*^ females appeared to store lower quantities of sperm, which could suggest that either the duration of copulation was reduced or that males produce less sperm. Given that when females were allowed to mate for an extended period (i.e. 24 h, 48 h, and 72 h), no difference in insemination rate was observed between *AeCyc*^*−/−*^ and wildtype, the lower sperm storage phenotype we observed in *AeCyc*^*−/−*^ within an hour could be due to a delayed start in sperm production. However, *AeCyc*^*−/−*^ mutant females appeared to store relatively less sperm, predominantly only in one or two spermathecal capsules compared to wildtype females in the extended mating experiments as well. This could explain the reduced hatching rates and lowered fitness.

Recent work in *Anopheles* demonstrated the direct connection between the endogenous circadian clock and mating behavior^48^. Knockdown of both *per* and *tim* in *An. gambiae* and *An. stephensi* reduces swarming behavior and insemination rates in the lab and under semi-field conditions. Furthermore, *per* and *tim* expression was shown to be higher in swarming vs non-swarming field-collected mosquitoes^48^. *Per* and *tim* also control the circadian rhythms of female mating activity in *Drosophila melanogaster*^49^. Moreover, gene transfer experiments implicate *per* in the species-specific behaviors of locomotor activity, love song rhythms, and time of mating^28,50^. *An. stephensi* swarming behavior is strongest when *per* and *tim* expression peaks^48^, which is not the case in *Aedes aegypti*, as shown in this study*.* While *per* expression is much reduced overall in *AeCyc*^−/−^ compared to wildtype, at least in female heads, *tim* expression is actually increased during the time the one-hour experiment was conducted. It is therefore not clear if the difference in expression level of these two genes could be responsible for the reduced mating success we observed. Another explanation might lie in the observation that mosquito antennae possess autonomous circadian clocks that could control circadian rhythms of olfactory response^42^. Because it has been suggested that *Aedes aegypti* males produces volatiles that attract females^51^, the sensitivity of *AeCyc*^*−/−*^ antennae to these compounds could be affected as well.

Finally, while the response to host odors and mating efficiency were reduced, the blood-feeding propensity of the *AeCyc*^*−/−*^ females was significantly higher than of the wildtype females throughout much of the day. The only exception was around ZT9 when the difference was not significant. Wildtype *Aedes aegypti* are at their most active at this time and their blood feeding propensity was highest at this point as well. Previously, it was shown that knockdown of *tim* expression reduces blood-feeding behavior in *Aedes aegypti*^13^, which seemed to suggest that deregulating the circadian clock reduces blood feeding propensity. This is in contrast to our observation. However, RNAi silencing of *tim, cry1* and *clk* in *An. gambiae* also increased blood feeding propensity^12^. As with the other behaviors it is not clear through which pathways the circadian clock controls blood feeding in mosquitoes, although a several other genes that impact blood feeding, such as odorant binding proteins, *takeout,* and others have been shown to be under circadian control^12^.

## Conclusions

Here we demonstrated the effect of disabling the endogenous circadian clock by knocking out the *cycle* gene on developmental processes, lifespan and essential behaviors in *Aedes aegypti*. *Cycle* knockout alter the cyclical expression patterns of several clock genes, with the most effect on *per* expression that becomes completely arrhythmic. Interestingly, *AeCyc*^*−/−*^ mutants maintain a diel activity throughout the light phase, but this too is strongly affected, as the characteristic bimodal activity peaks early and late during the light phase seen in wildtype are lost in *AeCyc*^*−/−*^ mutants. The largest impact of *cycle* knock out on life history traits is on embryonic development, as *AeCyc*^*−/−*^ have a much delayed hatching rate. Not surprisingly, various circadian behaviors were impacted by *cycle* KO as well. *AeCyc*^*−/−*^ show a reduced response to host odors, reduced mating success, but an increase in blood feeding propensity. Together with other recent work on the circadian clock of mosquitoes, this work contributes to elucidate the pathways through which the circadian clock controls mosquito behavior. Future studies aiming at understanding the potential impact of *cycle* and other clock genes KO on metabolism, insecticide susceptibility and vector competence, could provide important insights on the biology of this important disease vector to ultimately deploy potential control strategies that takes into account time-of-day parameters.

## Methods

All experiments methods were performed according to relevant guidelines and regulations for animal use and laboratory practices, including environmental health, occupational safety, and biosafety. All studies and facilities were approved by the Institutional Biosafety Committee (IBC# 2018-029) of Texas A&M University.

### Mosquito rearing

*Ae. aegypti* (Liverpool strain) were maintained at 27 °C, 60–70% relative humidity (RH) on a 12:12 h light/dark cycle (this includes 1 h dawn and 1 h dusk transitions). Eggs were hatched in deionized water, and larvae were fed ground Tetramin® fish food daily. Adults were provided with cotton balls soaked in a 10% sucrose solution. Colony mosquitoes were fed once a week on de-fibrinated calf or sheep blood (Hemostat Laboratories) fed through an artificial membrane feeder.

### *Ae. aegypti cycle* KO mutant generation

Six sgRNAs targeting exons 3 and 5 of *cycle* were designed using CHOPCHOPv2^52,53^. These sgRNAs were generated by in vitro transcription following Bassett et al.^54^. sgRNA cutting efficiency was tested in vitro on purified PCR product of the *cycle* target region using the Cas9 manufacturer's protocol (PNA Bio). An sgRNA for targeting sequence TCGTACACCGAGGGCCACTACAAGC in exon 5 (AAEL002049-RD) was selected for further injection. An injection solution was prepared by combining 100 ng/µL of sgRNA and 200 ng/µL of Cas9 in RNase-free water. This mixture was incubated at 37 °C for 20 min, and centrifuged at 4 °C for 30 min at 14,000×*g*. The injection mixture was kept on ice protected from direct light until the injection.

To collect embryos for injection, adult female *Ae. aegypti* were blood-fed de-fibrinated calf blood and maintained in the insectary without access to egg paper. After four days, ~ 30 adult female *Ae. aegypti* were placed into a 50 mL conical tube containing a wet cotton ball and a disc of wet filter paper and placed in the dark for 25 min to lay eggs. Freshly laid embryos were transferred to a clean wet filter paper and aligned with a paintbrush. Aligned embryos were transferred to coverslip by double-sided masking tape and covered in emersion oil for injection.

Needles were fabricated from borosilicate capillaries (1 mm × 0.5 mm × 10 cm) using the P-1000 Pipette Puller and beveled using the BV-10 Micropipette Beveller (Sutter Instrument). Injections were performed using a Pneumatic Picopump (World Precision Instruments) and visualized with the Dino-Lite Edge Digital Microscope (Model 7115MZTL) at 150 × magnification and a laptop computer running DinoXcope (Dunwell Technologies Inc.). Following injection, embryos were rinsed with distilled water and kept on wet filter paper for four days in the insectary before being placed in a water basin for hatching. Surviving larvae were raised to adulthood as previously described.

DNA from surviving adults was extracted using the NucleoSpin Tissue Kit (Macherey–Nagel) from a single leg using the Phire Animal Tissue Direct PCR Kit (Thermo-Fisher Scientific). PCR was conducted on extracted DNA using GoTaq® Flexi PCR Kit (Promega) or the Phire Animal Tissue Direct PCR Kit (Thermo-Fisher Scientific) using a fluorescent forward primer for fragment analysis. Fragment analysis was conduct on a 3500 Genetic Analyzer (Applied Biosystems) with the GeneScan™ 500 LIZ™ size standard (Thermo-Fisher Scientific) and analyzed in Geneious (Biomatters) to detect indels at the CRISPR/Cas9 target site. Confirmed mutants were outcrossed with wildtype mates for four generations before heterozygous *Cyc*^*−/*+^ individuals were inter-mated to generate the homozygous *AeCyc*^*−/−*^ knockout line.

### Endogenous clock gene expression

The expression of seven essential clock genes, *cycle* (*AeCyc)*, *clock (AeClk)*, *period (AePer)*, *timeless (AeTim)*, *cryptochrome-1*(*AeCry1*), *cryptochrome-2* (*AeCry2*) and *par domain protein 1* (*AePdp1*), was examined by qRT-PCR from heads of *AeCyc*^*−/−*^ and wildtype mosquitoes collected at four-hour time intervals across a single light/dark (LD) cycle, as well as across 24 h of darkness (DD) after entrainment to LD cycles (Table S1). LD and DD cycles both are recorded as Zeitgeber Time (ZT), in LD cycle particularly ZT0 being defined as the end of the 1 h dawn transition and the beginning of the full light cycle, and ZT12 defined as the time of lights off at the end of the dusk transition. mRNA was extracted from 10 female heads per replicate using the Dynabeads® mRNA DIRECTTM Micro Purification Kit (Thermo Fisher Scientific). RNA quantity and quality were assessed on a NanoDrop spectrophotometer, and RNA Pico LabChip runs on an Agilent BioAnalyzer 2100. qRT-PCR of *AeCyc*, *AePer*, *AeTim*, *AeCry1*, *AeCry2* and *AePdp1* were performed on 3 replicates using SYBR Green One-Step Real-Time RT-PCR (Thermo Fisher Scientific) on the Bio-Rad CFX96 Real-Time System. Normalization of genes were calculated relative to ribosomal protein S6, which has previously shown to have constitutive expression across the light/dark cycles in *Ae. aegypti*^5,8^.

### Luciferase transcriptional assay

The pGL-*dpPer*4Ep and pAC5.1V5/HisA plasmids were provided by Zhu et al.^55^ and pAC-*Renilla*-Luc control plasmid was provided by McDonald et al.^56^. pAC plasmids containing wildtype *AeClk, AeCyc*^*WT*^, and *AeCyc*^*−/−*^*,* were generated by PCR amplification from cDNA generated from the wildtype or mutant *AeCyc* mosquito lines that were subsequently subcloned into pAC5.1V5/HisA*.* Wildtype *AeClk* was subcloned between *Kpn*I and *Xho*I, wildtype *AeCyc* was subcloned between *EcoR*I and *Xba*I, and the mutant *AeCyc* was subcloned between *EcoR*I and *Xho*I. Details of primers used for the cloning are provided in Table S2.

S2 cells were maintained at 25 °C in Schneider's *Drosophila* medium (Gibco) supplemented with 10% heat-inactivated fetal bovine serum (FBS, Gibco) and 100 U/mL penicillin and streptomycin (Gibco). Transient transfections were performed as previously described^17,57^ in 12-well plates using 10 ng/well of *dpPer4Ep*-Luc as a reporter and 30 ng/well pAC-*Renilla*-Luc as a normalization control. S2 cells were co-transfected with 5 ng/well of pAC plasmids expressing wildtype *AeClk*, wildtype *AeCyc* and/or the mutant *AeCyc*. For luciferase assay, cells were lysed with 50 μl of 1X Passive lysis buffer (Promega). Firefly and renilla luciferase activities were quantified with a Dual-Luciferase reporter assay system (Promega) using 5 μl of cell protein lysate on a VICTOR3 V Multilabel Plate Counter (PerkinElmer). Firefly luciferase activity was computed relative to renilla luciferase activity.

### Locomotor activity assay

Males and females were raised together for 5 days and then briefly knocked down by cooling at 4 °C and individually placed into glass vials. One end of the glass vial was blocked with a cotton ball and the second end of the glass vial was blocked with a plastic tube containing 10% sugar water and a fabric wick that enters the glass vial. Glass vials were placed in the Locomotor Activity Monitor 25 (Trikinetics). The activity monitor was placed inside an incubator with an DEnM Environmental Monitor (Trikinetics) kept at 27 °C and 60–70% relative humidity. Locomotor activity of wildtype and *AeCyc*^*−/−*^ mosquitoes was quantified by infrared beam breaks in the activity monitor which was collected by the accompanying DAMSystem3 software. The first 24 h of activity monitor data were discarded. Data was collected for an additional eight days. On the first two days, mosquitoes were exposed to a 12:12 light–dark cycle, followed by three days of darkness, and three subsequent days of 12:12 light/dark cycle.

### Measurement of life history parameters

#### Egg hatching

Mosquito eggs were collected from 25 five-days old wildtype and *AeCyc*^*−/−*^ females they were mated with respective genotype males. Freshly laid eggs were collected, and 25 eggs were randomly chosen and placed in water containing fish food for hatching. This was performed for six replicates. Newly hatched larvae were counted for 30 days.

#### Pupation rate

Six replicates of 25 freshly hatched first instar wildtype and *AeCyc*^*−/−*^ larvae (L1) were collected and placed into a small container with 25 mL of water containing fish food ad libitum. The number of pupae was recorded daily.

#### Adult emergence

Six replicates of 25 new wildtype and *AeCyc*^*−/−*^ pupae were collected from wildtype and *AeCyc*^*−/−*^ pans. Each replicate was placed into a small plastic cup with water in an adult cage. The number of emerged adults was recorded daily.

#### Longevity/survivorship

For each wildtype and *AeCyc*^*−/−*^ genotype, males and females were raised together for 5 days and allowed to mate. Mosquitoes were then briefly knocked down by cooling at 4 °C and five replicates of 30 adult male or female wildtype or *AeCyc*^*−/−*^ mutants were placed into adult bins, and survivorship was recorded for 30 days.

### Mating success

Males and females were separated during the pupal phase. Three replicates of 50 five-days old adult females and males were placed in 30 × 30 × 30 cm cages and allowed to mate for 1 h, 24 h, 48 h, and 72 h. The 1 h experiment was conducted towards the end of the light phase, i.e., during the peak in locomotor activity of wildtype *Ae. aegypti*. Insemination experiments were performed both within wildtype and *AeCyc*^*−/−*^ lines, as well between the two strains. At the end of each experiment, mosquitoes were killed and their spermathecae examined at 400X magnification to determine insemination status.

### Attraction to human odor

Adult female mosquitoes were raised in the presence of males for five days. One day prior to wind tunnel experiments, four replicates of 50 female mosquitoes were briefly knocked down by cooling at 4 °C and 50 females were sorted into the release chamber. A cotton ball soaked in 10% sugar water was provided after sorting. Twelve hours before the wind tunnel experiment, the cotton ball soaked in sugar water was replaced with a cotton ball soaked in fresh water. Three hours prior to olfactometer experiments, the holding chamber containing mosquitoes was moved from the insectary to the wind tunnel room, which was kept at 27–30 °C and 70–80 RH. To provide a human odor source, a white sock worn for two days by a volunteer was incubated at 37 °C for 3 h.

The dual choice olfactometer measures 6ft × 2.5ft × 2.5ft. Warm humidified air was released into the olfactometer through two odor ports at a speed of ~ 0.5 m/s. The incubated sock was placed in one of the two collecting chambers connected to the odor ports, and the other collecting chamber was left empty. Position of the sock in left vs right collection chamber was switched between experimental days. CO_2_ (5%) was released into the olfactometer from a position in between the two odor ports. Experiments conducted during the dark phase were conducted at ~ 5 lx.

After opening the release cage, experiments were run for 20 min. At that point the number of mosquitoes that left the holding chamber were counted. These were classified as “active”. Additionally, the number of mosquitoes entering one of the two odor ports were counted and classified as “responsive”. Experiments were run at three different times: 6 h into the light phase (Zeitgeber time 6; ZT6), 12 h into the light phase (ZT12), and 6 h into the dark phase (ZT18).

### Blood feeding activity

To assess the blood feeding behavior of the *AeCyc*^*−/−*^ mutant mosquitoes, a series of assays were performed in LD conditions. The blood-feeding assays were repeated at least four independent time points, with three replicates (20 mosquitoes in each replicate). 5–6 days old, inseminated females of both wildtype and *AeCyc*^*−/−*^ mutants were allowed to blood feed at four-hour intervals across the light/dark condition (i.e., ZT1, ZT5, ZT9, ZT13 and ZT17). Defibrinated sheep blood at 37 °C, which we routinely use for colony maintenance, was provided through an artificial membrane feeder. Mosquitoes were allowed to blood feed for 20 min, after which they were knocked down by cooling at 4 °C and scored as nonblood-fed, partially blood-fed, or fully engorged under a microscope. A total percentage of blood-fed *AeCyc*^*−/−*^ females were compared with wildtype females at different time points.

### Statistical analysis

Endogenous clock gene expression data were analyzed using One-Way ANOVA and Post Hoc Bonferroni tests were used for pair-wise comparisons between *AeCyc*^*−/−*^ and wildtype at different time points during both LD and DD cycles. Luciferase assay data was analyzed using One-Way ANOVA by comparing *AeClk*:*AeCyc*^*−/−*^ vs. *AeClk*:*AeCyc*^*WT*^*.* Mosquito activity and host-seeking behavior in the wind tunnel were analyzed by generalized linear mixed models. Data were fit to a binomial distribution. Replicates were used as a random variable. Genotype was used as the independent variable and the level of activity and responsiveness were used as the response variable. Egg hatching rate, pupation rate, emergence rate and mosquito circadian activity were compared between wildtype and *AeCyc*^*−/−*^ mutants using the Welch two sample t-test. Survivorship was analyzed using the Cox proportional hazards regression model using the R package “survival”. Blood feeding behavior at different time points during LD cycle was statistically analyzed using Student t-tests. All the statistical analyses were performed using R software (version 3.6.3).

## Supplementary Information

Supplementary Tables.

## Data Availability

All the data pertaining to knockout line creation, gene expressions, all the behavioral studies and statistical analysis were included in the supplementary pages S1.
