# Supplementary material for: Impact of disabled circadian clock on yellow fever mosquito Aedes aegypti fitness and behaviors
Source: Sci Rep. 2022 Apr 27;12:6899. doi: 10.1038/s41598-022-10825-5 (PMC9046260; doi:10.1038/s41598-022-10825-5)
Supplement: Supplementary file 1 — Supplementary Tables. [file 41598_2022_10825_MOESM1_ESM.dotx]

**Table S1**

| **Gene** | **Gene code** | **Primers (F: forward; R: reverse)** |
| --- | --- | --- |
| *Clock* | AAEL022593 | F: 5’-CCGACGACAACGACTACAAA-3’  R: 5’-GTTCGGATTGCACAGTTCCT-3’ |
| *Cycle* | AAEL002049 | F: 5’-GAGCAGTTGTCTTCTTCGGATT-3’  R: 5’-CACAGCCTTGTCACACCTTG-3’ |
| *Period* | AAEL008141 | F: 5’-GGAGCAATTCGTCCGGTAG-3’  R: 5’-TGGGTTCAACAGACGTTTTC-3’ |
| *Timeless* | AAEL019461 | F: 5’-GGCTTTCTCGTACAAGAGCTTC-3’  R: 5’-GAACTTCAGGAAATAGGTCA-3’ |
| *Par domain protein1* | AAEL005255 | F: 5’-GACGCATGAAGGAGAATCAA-3’  R: 5’-TGGGTTCAACAGACGTTTTC-3’ |
| *Cryptochrome-1* | AAEL004146 | F: 5’-GCCGACGCTCTATGTCCACG-3’  R: 5’-CGATCATCGGTGCCGGATAGTC-3’ |
| *Cryptochrome-2* | AAEL002602 | F: 5’-GCAACTACGCCACAGTACGA-3’  R: 5’-GCTTCCGTTTCACCTCTCAC-3’ |

**Table S2**

| **Wildtype *AeClk* was subcloned between *Kpn*I and *Xho*I using the following primers (F, forward; R: reverse)** |
| --- |
| F: 5’-TCGGGGTACCATGGAGGATGATCCGGATG-3’  R: 5’-TAGACTCGAGCATATCGCTCCTGGGGTGAC-3’ |
|  |
| **Wildtype *AeCyc* was subcloned between *EcoR*I and *Xba*I using** |
| F: 5’-GGTGGAATTCATGATCTATTCAAAAAGTGCG-3’  R: 5’-CCCTCTAGACTCGACGAGTGGTAACGGC-3’ |
|  |
| ***AeCyc^-/-^* was subcloned between *EcoR*I and *Xho*I using** |
| F: 5’-GGTGGAATTCATGATCTATTCAAAAAGTGCG-3’  R: 5’-TAGACTCGAGGCTCCTGGTCGGAGAGG-3’ |

**Table S3a**

The percentage of non-fed partially fed and fully fed mosquitoes in all the different time points are shown.

The standard error and the p-values (t-test) are also shown.

| **Mosquito fed at different time points (ZT)** | **Types of mosquito** | **Percentage of mosquitoes** | | | **Standard error** | | | **p-value (T-test) of total fed (with respect to wildtype)** |
| --- | --- | --- | --- | --- | --- | --- | --- | --- |
|  |  | **Non-fed** | **Partially**  **fed** | **Fully fed** | **Non-fed** | **Partially**  **fed** | **Fully**  **fed** |  |
| **1** | ***AeCyc^-/-^*** | 39 | 15 | 46 | 2.95 | 1.73 | 1.30 | < 0.05; Significant |
|  | **WT** | 64 | 8 | 28 | 1.79 | 0.55 | 1.52 |  |
| **6** | ***AeCyc^-/-^*** | 33 | 15 | 57 | 4.04 | 1.00 | 3.65 | < 0.05; Significant |
|  | **WT** | 56 | 12 | 26 | 1.79 | 0.89 | 1.10 |  |
| **11** | ***AeCyc^-/-^*** | 25 | 18 | 57 | 2.24 | 1.14 | 2.07 | > 0.05;  Non-significant |
|  | **WT** | 36 | 18 | 46 | 1.48 | 2.19 | 1.92 |  |
| **16** | ***AeCyc^-/-^*** | 53 | 9 | 38 | 2.30 | 0.84 | 2.07 | < 0.05; Significant |
|  | **WT** | 82 | 4 | 14 | 0.55 | 0.84 | 0.84 |  |
| **21** | ***AeCyc^-/-^*** | 79 | 8 | 14 | 1.48 | 1.52 | 1.30 | < 0.05; Significant |
|  | **WT** | 94 | 2 | 3 | 1.30 | 0.55 | 0.89 |  |

**Table S3b**

**Independent t-test between Cyc and WT groups at different time points.**

The assumption of homogeneity of variances was tested and satisfied via Welch two sample t-test.

| **Mosquito fed at different time points (ZT)** | **Types of mosquito** | **t-test for Equality of Means** | | |
| --- | --- | --- | --- | --- |
|  |  | **t** | **df** | **Welch’s t-test**  **(P-value)** |
| 1 | *AeCyc^-/-^* | 3.24 | 6.59 | 0.015 (Significant) |
|  | *WT* |  |  |  |
| 5 | *AeCyc^-/-^* | 5.1 | 4.81 | 0.003 (Significant) |
|  | *WT* |  |  |  |
| 9 | *AeCyc^-/-^* | 1.83 | 6.94 | 0.109 (Non-significant) |
|  | *WT* |  |  |  |
| 13 | *AeCyc^-/-^* | 5.48 | 4.45 | 0.003 (Significant) |
|  | *WT* |  |  |  |
| 17 | *AeCyc^-/-^* | 5.013 | 7.86 | 0.001 (Significant) |
|  | *WT* |  |  |  |
